# Direct Translocation as Major Cellular Uptake for CADY Self-Assembling Peptide-Based Nanoparticles

**DOI:** 10.1371/journal.pone.0025924

**Published:** 2011-10-05

**Authors:** Anna Rydström, Sébastien Deshayes, Karidia Konate, Laurence Crombez, Kärt Padari, Hassan Boukhaddaoui, Gudrun Aldrian, Margus Pooga, Gilles Divita

**Affiliations:** 1 Centre de Recherches de Biochimie Macromoléculaire, CRBM-CNRS, UMR-5237, UM1-UM2, Department of Molecular Biophysics and Therapeutics, University of Montpellier, Montpellier, France; 2 Department of Developmental Biology, Institute of Molecular and Cell Biology, University of Tartu, Tartu, Estonia; 3 Institut National de la Santé et de la Recherche Médicale (INSERM) U1051, Institut des Neurosciences de Montpellier, Montpellier, France; University of Cincinnati, United States of America

## Abstract

Cell penetrating peptides constitute a potent approach to overcome the limitations of in vivo siRNA delivery. We recently proposed a peptide-based nanoparticle system, CADY, for efficient delivery of siRNA into numerous cell lines. CADY is a secondary amphipathic peptide that forms stable complexes with siRNA thereby improving both their cellular uptake and biological response. With the aim of understanding the cellular uptake mechanism of CADY:siRNA complexes, we have combined biochemical, confocal and electron microscopy approaches. In the present work, we provide evidence that the major route for CADY:siRNA cellular uptake involves direct translocation through the membrane but not the endosomal pathway. We have demonstrated that CADY:siRNA complexes do not colocalize with most endosomal markers and remain fully active in the presence of inhibitors of the endosomal pathway. Moreover, neither electrostatic interactions with cell surface heparan sulphates nor membrane potential are essential for CADY:siRNA cell entry. In contrast, we have shown that CADY:siRNA complexes clearly induce a transient cell membrane permeabilization, which is rapidly restored by cell membrane fluidity. Therefore, we propose that direct translocation is the major gate for cell entry of CADY:siRNA complexes. Membrane perturbation and uptake are driven mainly by the ability of CADY to interact with phospholipids within the cell membrane, followed by rapid localization of the complex in the cytoplasm, without affecting cell integrity or viability.

## Introduction

Small interfering RNA (siRNA) has great potential as a therapeutic molecule, due to its high target specificity, efficiency for gene silencing and its simple design [1], [2]. However, the major limitation for clinical development of siRNA, remains its low bioavailability and poor cellular uptake associated with the lack of permeability of the cell membrane to negatively charged nucleic acids [3]–[5]. Therefore, the success of siRNA is dependent on carrier molecules and numerous non-viral strategies have been proposed to improve the delivery of synthetic small oligonucleotides [6]–[9]. During the last decade, Cell-penetrating peptides (CPPs) have been widely used for the delivery of therapeutic molecules and have been reported to favour the delivery of a large panel of cargos (plasmid DNA, oligonucleotide, siRNA, PNA, protein, peptide, liposome, nanoparticle…) into a wide variety of cell types and in vivo models [10]–[12]. CPPs can penetrate biological membranes and introduce biomolecules across the plasma membrane into the cytoplasm, improve their intracellular routing, thereby facilitating interactions with the target. CPPs can be subdivided into two main classes, the first requiring chemical linkage with the cargo and the second involving the formation of stable, non-covalent complexes [10]–[12].

Since the discovery of the first CPPs about 20 years ago, several mechanisms for their cellular uptake have been proposed [13], [14]. Today it has become clear that there is no universal pathway of cell entry, but rather that it depends on the physical properties of the CPP together with factors such as the nature of the cargo, the concentration used and the presence of specific heparan sulfate proteoglycans (HSPGs) on the cell surface. For most CPPs, evidence for several routes has been reported, dependent or not on the endosomal pathway [10], [13]–[15]. In most cases, the first contacts between CPPs and the cell surface occur through electrostatic interactions with components of the extracellular matrix, cell surface proteoglycans, followed by a remodelling of the actin network and a selective activation of small GTPases [14], [16], [17]. These interactions constitute the ‘onset’ of internalization and have a major impact on membrane fluidity, thereby promoting CPP cell entry via macropinocytosis [18], clathrin-dependent endocytosis [19], or via membrane perturbation [20]–[22]. Each mechanism has its own liabilities. Uptake via endocytosis, as seen for numerous CPPs such as Tat, Arg9, Transportan and Penetratin, may hamper biological activity due to the fact that a large proportion of CPP-cargo is trapped in endosomal compartments and then degraded in the lysosomes. At higher concentrations, starting from 10 µM, TAT, Penetratin and Arg9 CPPs have been shown to enter the cell via direct penetration, which can induce irreparable membrane damage and cell death [15], [20], [22].

In order to improve cellular uptake of charged oligonucleotides, we have developed an alternative non-covalent strategy for the delivery of siRNA, based on amphipathic peptides, that has been reported to improve siRNA delivery *ex-vivo* into a large panel of cell lines and *in vivo* [23], [24]. Non-covalent strategies appear to be more appropriate for siRNA delivery, and yield significant associated biological response [25]–[27]. We have recently described a new peptide-based delivery system for siRNA using the secondary amphipathic peptide CADY [24]. CADY is a 20-residue peptide, “Ac-GLWRALWRLLRSLWRLLWKA-cya”, that binds non-covalently to siRNA by combining both electrostatic and hydrophobic interactions to form stable nano-complexes that can enter cells, seemingly independently of endocytosis. We demonstrated that CADY efficiently delivers siRNA in various cell lines providing a knockdown response at low nanomolar range [24], [28]. A better understanding of the uptake mechanism of CADY:siRNA complex is essential in order to further develop this technology for in vivo and clinical evaluation.

In the present work, the cellular uptake mechanism of CADY:siRNA particles has been investigated in detail by combining biochemical and microscopic approaches. Using confocal microscopy as a non-invasive and descriptive approach, we report that CADY:siRNA does not colocalize with endosomal markers suggesting non-endosomal cellular uptake of the complexes. These results were further confirmed by transmission electron microscopy. Moreover we show that although there is an interaction of CADY:siRNA complex with heparan sulphates, this interaction is not required for cellular entry. However, monitoring changes in intracellular calcium levels with the calcium indicator FURA, reveals that CADY:siRNA complexes induce a transient permeabilization of the cell membrane. Taken together, these results suggest that the main mechanism of cell entry is driven by interactions between CADY and cell membrane phospholipids and occurs via membrane perturbation, followed by rapid release in the cytoplasm, without disrupting cellular integrity or biological viability.

## Results

### Interaction with cell surface-heparan sulphates is not required for CADY cellular uptake

**Figure 1:**
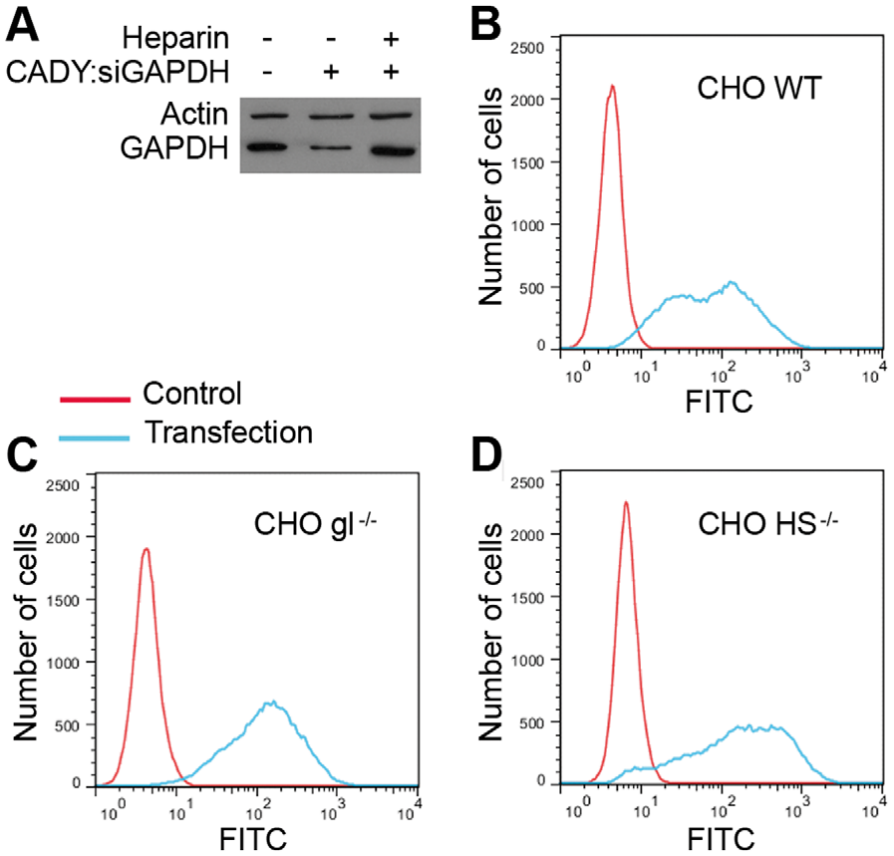
Electrostatic interaction between CADY and HSPGs is not required for efficient transfection. (**A**) HeLa cells were pre-treated for 30 min with free medium or medium containing 5 µg/ml heparin prior to addition of 80 nM siRNA complexed to CADY at a 1∶40 molar ratio. Cells were incubated in the presence of heparin for 1 hr, then extensively washed in PBS and replaced in DMEN containing 10% FCS. Cells were harvested after 48 hours and protein levels were analysed by Western blotting. CHO wild type (WT) (**B**), glycosaminoglycan deficient (gl^−/−^) (**C**) and heparan sulfate deficient (HS^−/−^) (**D**) cells were transfected with 80 nM FITC-labeled siRNA complexed to CADY at a 1∶20 molar ratio. After 1.5 hrs, cells were extensively washed, trypsinized and analyzed by FACS.

The cell membrane is covered with negatively charged proteoglycans, such as heparan sulphate proteoglycans (HSPGs) which constitute the first external barrier before the lipid phase of the membrane. For numerous CPPs, the first step of cellular uptake mechanism has been reported to be driven by the electrostatic interaction between the cationic CPPs and the anionic HS-component of the proteoglycan [10], [14]. We have investigated if this interaction was also required for cellular uptake of the CADY/siRNA complex. As reported in Figure 1A, the presence of the HSPG analogue heparin (5 µg/ml), during the transfection of HeLa cells with CADY:siRNA complexes, at molar ratio 40/1, (CADY and siRNA concentrations of 3.2 µM and 80 nM, respectively), clearly reduced the GAPDH target protein knock down, suggesting that the heparin analogue sequestered CADY:siRNA complexes. This result confirms our previous results, showing that *in vitro*, CADY:siRNA complexes can interact with HSPGs, implying a possible initial electrostatic contact at the cell surface [28]. However, we cannot exclude that the CADY:siRNA complexes are not only sequestered by heparin, but also dissociated, as shown for higher HSPG concentrations [28]. To better address this question, transfection and cellular uptake efficiencies of CADY:siRNA were compared for three different CHO cell lines: CHO^WT^, CHO^HS−/−^ lacking heparan sulphates, and CHO gl^−/−^ lacking glycosaminoglycanes (GAGs). The three cell lines were transfected with CADY:siRNA-FITC at molar ratio 20/1 (CADY and siRNA concentrations of 1.6 µM and 80 nM, respectively). Analysis of cellular uptake by FACS, revealed a similar transfection efficiency of ∼90% for all three cell types (Figure 1B–D), leading us to conclude that, in contrast to several CPPs [14], [19] the cellular uptake of CADY:siRNA complexes is not mediated by electrostatic interactions with GAG components. Due to it positive net charge CADY:siRNA complexes can interact with HSPG, an interaction which is not required for cellular uptake, but which partially dissociate the CADY:siRNA complex.

### CADY-mediated siRNA delivery is independent of endocytosis

**Figure 2:**
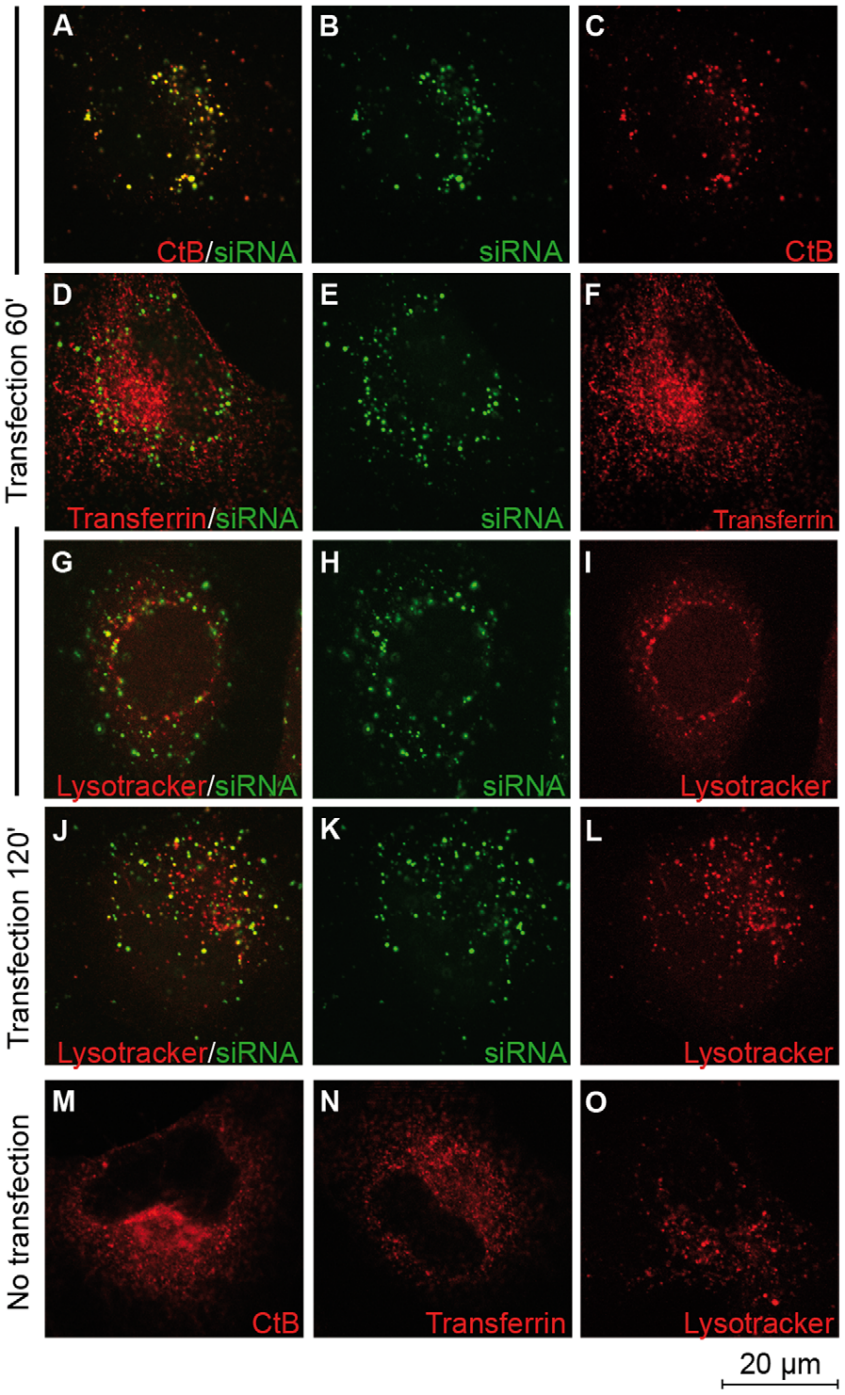
Cellular uptake of CADY:siRNA-FITC complexes in the presence of endosomal and lysosomal markers. HeLa cells grown on glass coverslips were transfected with 80 nM FITC-labeled siRNA complexed to CADY at a 1∶4 ratio together with either choleratoxin subunit B (CtB) (**A–C**), transferrin (**D–F**) or lysotracker (**G–L**). Cells were extensively washed and fixed in PFA at indicated time points. (**M–O**) Non-transfected HeLa cells treated with CtB, Transferrin and Lysotracker.

**Figure 3:**
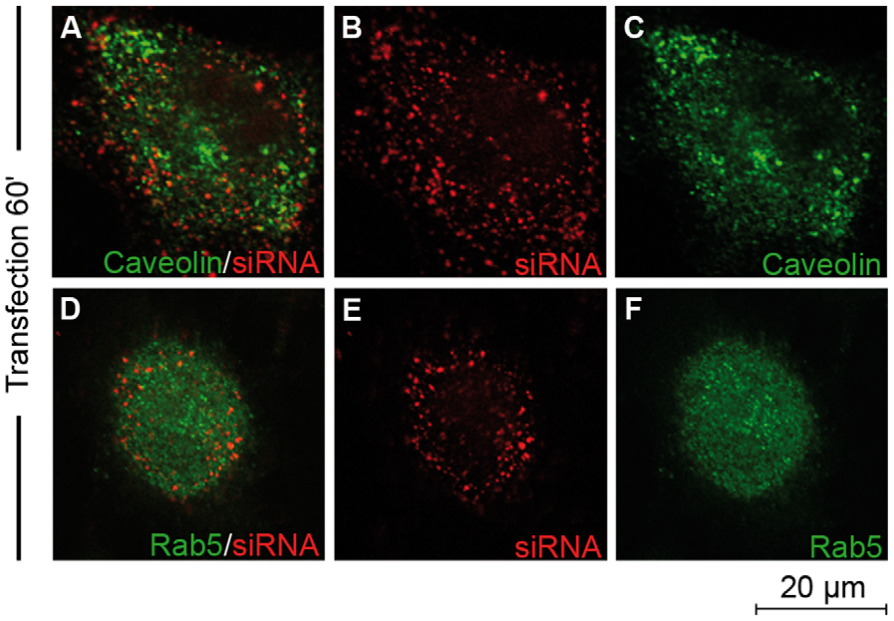
Cellular uptake of CADY:siRNA-Cy3 complexes in the presence of endosomal markers. HeLa cells grown on glass coverslips were transfected with 80 nM Cy3-labelled siRNA complexed to CADY at a 1∶4 molar ratio. After 1 hr, cells were fixed in PFA and stained with anti-caveolin (**A–C**) or anti-Rab5 (**D–F**).

We previously reported that the presence of the endosomal inhibitors amiloride, nocodazole or methylbetacyclodextrin did not affect the efficacy of CADY to deliver siRNA [24]. Previous results shown that CADY:siRNA complexes are efficiently taken up by cells in the presence of endocytotic inhibitors. However, one should keep in mind that none of these inhibitors are 100% specific for one pathway. Furthermore, there is always a risk that when inhibiting one pathway, the cells try to compensate by activating another pathway [29], [30]. To circumvent this problem, we investigated whether CADY transfected siRNA was trapped in endocytotic vesicles and colocalize with endosomal markers by confocal microscopy. HeLa cells were transfected with 80 nM fluorescently labelled siRNA (FITC- or Cy3-siRNA) in complex with 320 nM CADY, in the presence of markers for different endocytotic pathways To follow the kinetics of endosomal uptake, fixed and unfixed cells were analyzed 20, 40, 60 or 120 minutes after transfection. Rhodamine-labelled transferrin and Lysotracker were used as a marker for clathrin-mediated endocytosis and lysosomes, respectively [31]–[32]. Anti-Rab5 was used as a general endosomal marker and anti-caveolin as a marker for caveolin-mediated endocytosis [32]–[35]. Similar results were obtained for fixed and unfixed cells. As reported in Figure 2 D–E and Figure 3 D–F (data not shown), neither transferrin, nor Rab5-positive endosomes co-localised with CADY:siRNA during the time points observed. Lysotracker co-localized with siRNA-FITC to some extent after 60 and 120 minutes (Figure 2 G–L). After 60 minutes of transfection, in the majority of the cells siRNA-Cy3 did not co-localize with caveolae-positive endosomes. (Figure 3 A–C) That siRNA/caveolae colocalization could be observed to a small extent, fits with previous results, indicating that a small fraction of CADY:siRNA uptake could be mediated by caveolae [24].

Cholera toxin subunit B (CtB) was used as a marker for lipid rafts. Although, it has been proposed to be taken up by both clathrin- and caveolin mediated endocytosis [33], [36], there is also uptake of lipid rafts by less defined endocytotic pathways [34]. As reported in Figure 2 A–C, Alexa594-labelled CtB co-localized to a large extent with siRNA-FITC between 20 and 120 minutes after transfection. However, when CtB was taken up in the absence of CADY:siRNA much smaller entities were observed with different distribution (Figure 2M). This implies that the co-localisation could be due to a CADY-mediated uptake of CtB. In contrast, cell entry of transferrin and lysotracker is not affected by the presence of CADY:siRNA. (Figure 2 N,O). Taken together; these data further support an uptake mechanism largely independent of classical endocytosis.

### CADY mediates siRNA-transfection independently of the cellular energy state

**Figure 4:**
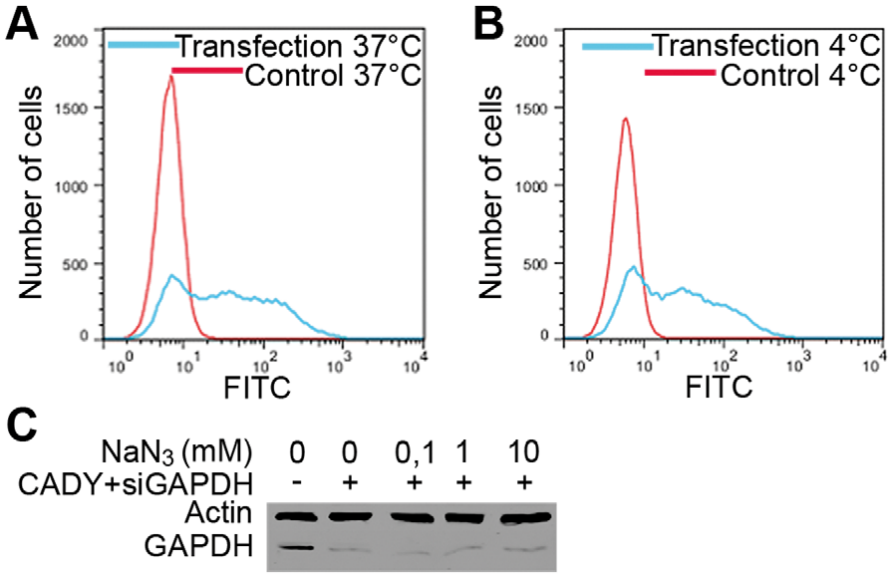
CADY transfection in energy depleted cells. HeLa cells were pre-incubated for 30 min at 37°C (**A**) or 4°C (**B**) prior to addition of 80 nM FITC-labeled siRNA complexed to CADY at a 1∶20 molar ratio. After 1.5 hr incubation at indicated temperatures, cells were washed, trypsinized and analysed by FACS. (**C**) HeLa cells were pre-treated with medium containing variable concentrations of sodium azide (NaN_3_) for 30 mins prior to addition of 80 nM siRNA complexed to CADY at a 1∶40 molar ratio. Cells were incubated for 1 hr in the presence of NaN_3_, then extensively washed, trypsinized and resuspended in medium containing 10% FCS. Cells were harvested after 48 hours and protein levels were analyzed by Western blotting.

The energy status of the cell plays an essential role for efficient intracellular translocation of several polycationic CPPs. Indeed, it has been shown for several CPPs that their transport through the plasma membrane is dependent on the transmembrane potential [21], [37]–[41]. To address these questions, the impact of temperature and of sodium azide, an inhibitor of mitochondrial oxidative phosphorylation, was evaluated on CADY transfection efficiency. HeLa cells were incubated with 80 nM FITC-labelled siRNA complexed with 1.6 µM CADY at 37°C and 4°C, for 90 min, cells were then trypsinized and the transfection efficiency was quantified by FACS. At 4°C, 60% of the cells were FITC positive, only slightly less than at 37°C (69% positive cells), demonstrating that metabolically inactive cells are efficiently transfected by CADY (Figure 4 A,B). In none of the conditions, (37° or 4°C) the cellular uptake of CADY:siRNA was associated with increased toxicity, as no major differences were observed in the level of PI positive cells after treatments (Figure S1). To further determine if CADY uptake was energy-dependent, HeLa cells were incubated with increasing concentration of sodium azide (ranging from 0.1 to 10 mM), 30 minutes prior to, and 1 hour after transfection with CADY:siGAPDH (3.2 µM and 80 nM, respectively), then the level of GAPDH protein was quantified by Western blotting after 48 hr. Both in the absence and in the presence of sodium azide, the levels of GAPDH were equally reduced demonstrating that the transfection efficiency was unaffected by the presence of sodium azide and that the cellular uptake of CADY:siRNA complexes does not depend on the energy state of the cell (Figure 4C).

### CADY:siRNA complex induces transient permeabilization of the plasma membrane

**Figure 5:**
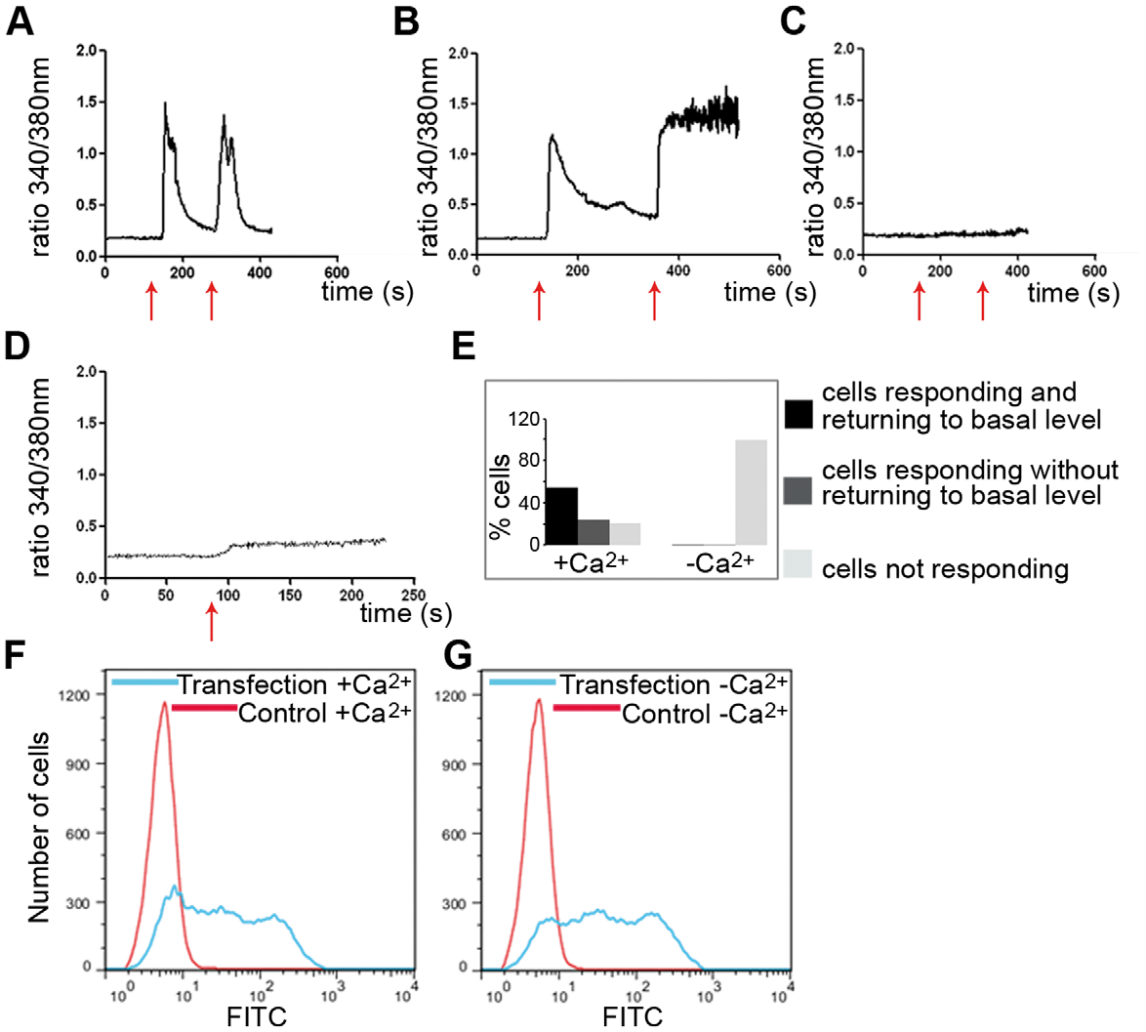
CADY:siRNA complexes induce cellular influx of calcium. HeLa cells were incubated with 5 µM FURA-2AM followed by washes in HEPES-Krebs-Ringer (HKR) buffer (**A–C,E**) or HKR buffer without calcium (**D,E**). The ratio of 340_(Ca-bound FURA-2AM)_/380_(FURA-2AM)_ nm excitatory wavelengths was recorded upon addition of 20 nM siRNA complexed to CADY at a 1∶20 molar ratio. HeLa cells were transfected with 80 nM FITC labeled siRNA complexed to CADY at a 1∶20 molar ratio in the presence (**F**) or absence (**G**) of calcium. After 90 mins, cells were washed, trypsinized and analysed by FACS.

We previously demonstrated that *in vitro* CADY:siRNA complexes strongly interact with phospholipid monolayers [28], implying that CADY:siRNA complexes can cross the plasma membrane by direct penetration. To test this hypothesis at the cellular level, we evaluated the fluctuations of intracellular calcium during CADY transfection using HeLa cells charged with the ratiometric fluorescent calcium indicator FURA-2AM. Considering that the concentration of calcium is lower inside the cell than outside, permeabilization of the plasma membrane, is associated with an influx of calcium. Once the cell has repaired the membrane, the calcium level is restored by active export [42]. 55% of the observed cells exposed to a 10 second pulse of CADY:siRNA complexes (1200 nM and 20 nM, respectively) responded by an immediate influx of calcium (Figure 5 A,E). The response was transient and calcium levels were fully restored after 100 seconds. Cells could be pulsed again, yielding the same response, indicating that the plasma membrane could easily be resealed, thereby ensuring cellular integrity and viability. 24% of the observed cells did not return to basal calcium levels after repeated pulses during the time of observation (Figure 5 A,E). It is possible that these cells needed longer time to restore their calcium levels, or that they did not recover after repeated exposure. 21% of the observed cells did not respond and remained with low basal calcium levels (Figure 5 C,E). To verify that the increase in intracellular calcium was the result of an extracellular influx not that of intracellular release from the endoplasmic reticulum, cells were loaded with FURA-2AM followed by extensive washes and incubation in calcium-free medium. Under these conditions, no increase in intracellular calcium was observed upon treatment with CADY:siRNA complexes (Figure 5 D,E). To exclude the possibility that cells showed no calcium influx due to decreased transfection efficiency in the absence of calcium, HeLa cells were transfected with FITC-labelled siRNA in the presence or absence of calcium. 90 min after transfection cells were trypsinized and the transfection efficiency was quantified by FACS. As reported in Figure 5 F–G no difference was observed between cells transfected with calcium (77% of FITC positive cells) and without calcium (79% of FITC positive cells).

### CADY:siRNA complex cellular uptake through direct translocation

**Figure 6:**
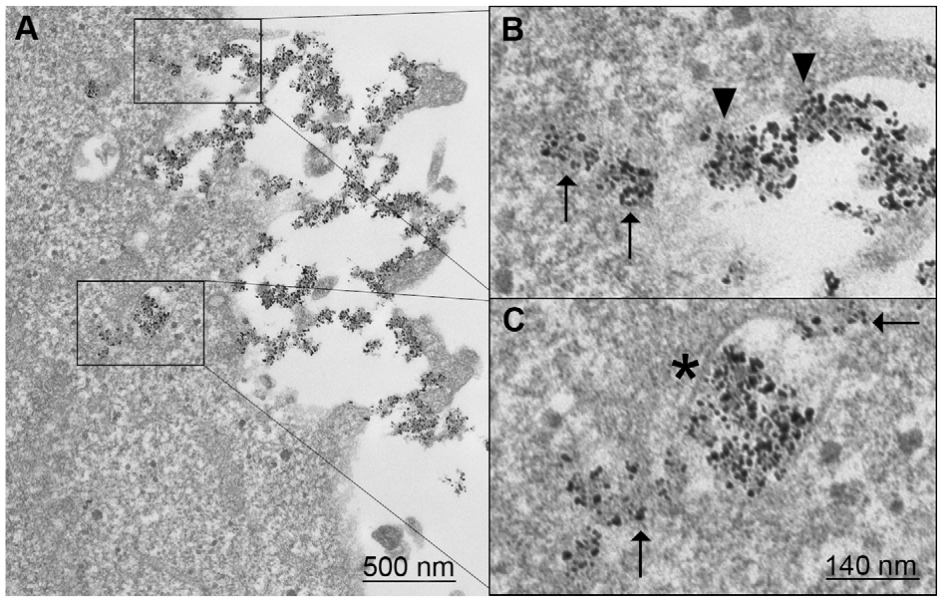
Electron micrographs of membrane interaction and internalization of CADY:siRNA nanoparticles. HeLa cells were incubated with complexes of nanogold-labeled siRNA (80 nM) complexed to CADY at a 1∶20 molar ratio for 2 h. Interaction of CADY:siRNA-nanogold complexes as dense particles with plasma membrane (**A, arrowheads in B**), translocation into cells (**arrows in B**) and localization in endosomal vesicle (**asterisk in C**) or free in cytosol (**arrows in C**).

In order to better understand the mechanism behind the membrane permeabilization observed by FURA labelling and to directly visualize cellular uptake of CADY:siRNA complexes, transfected cells were analyzed by transmission electron microscopy. HeLa cells were transfected with CADY:siRNA-gold (molar ratio 20/1, with concentrations of 1.6 µM and 80 nM respectively) during 30 minutes or 2 hours prior to fixation, Gold-labelled CADY:siRNA nanoparticles avidly associated with cells and after 30 minutes were bound with the cell surface as well as inside the cell, and after 2 hours more nanoparticles were found accumulating in the cytoplasm (Figure 6A and data not shown). The particles were not observed to induce endocytosis at the cell surface. Each nanoparticle contains several siRNA molecules, and although the CADY peptide is not visible, we can estimate each particle to be around 100 nm (indicated by arrows in Figure 6B and C), in line with our previous observations by dynamic light scattering [28]. There is a clear disorganisation of the cell membrane, seemingly due to the high affinity of CADY:siRNA particles for phospholipids. In closer detail, particles interacting directly with the cell membrane were identified (arrowheads in Figure 6B), as well as internalized particles in the cytoplasm with no surrounding membrane (arrows in Figure 6B). However, a small fraction of the nanoparticles, often grouped together in larger complexes, was found inside large vesicles (asterisk in Figure 6C), indicating that there can be a difference in the uptake depending on the size of the particles. Interestingly, the CADY:siRNA particles seemed to interact with the vesicle membrane as it often was not intact, and with several surrounding complexes on the outside. These data support the previous observations of a non-endocytic and direct penetrating mechanism of CADY.

## Discussion

Understanding cellular uptake of cell penetrating peptides remains a major task in order to improve their potency and promote their *in vivo* application. During the last decade extensive studies have revealed that several factors including the nature and the structure of the CPP, their mode of application (covalently-linked or self-assembled with the cargo), the nature of the cargo, play a major role in the cellular uptake mechanism [10]–[15]. Today, it is clearly established that different cell entry routes can existed simultaneously or sequentially depending on the CPP. In the present work, we have investigated in detail, the cellular uptake mechanism of the secondary amphipathic peptide CADY, a potent non covalent carrier for siRNA delivery [24]. In many cases, analysis of CPP cellular uptake has been performed with labelled-CPP in the absence of the cargo, therefore results can be misguiding. We have focused on the cellular behavior of the cargo upon CPP-delivery, following fluorescently labeled-siRNA within the cell and their associated biological response, rather than monitoring free CADY molecule. We propose that the major route for CADY:siRNA cell entry occurs by direct membrane translocation rather than by endocytosis, followed by a rapid release of the siRNA within the cytoplasm.

For several CPPs the first contact with the cell surface involves interactions with HSPG components that trigger cellular uptake. In contrast, in the case of CADY:siRNA, although the complex is able to interact with heparan sulphates, this interaction is not required for cell entry, as also suggested for other CPPs [43]. However, the presence of HS can alter the stability of the particles and reduce efficiency at low complex molar ratios (5/1; 10/1), but can also result in the dissociation of large particles and limit aggregation at the cell surface as confirmed by EM measurements.

CADY:siRNA complex does not follow an endosomal route. Indeed, siRNA delivered into the cells by CADY does not co localize with classical endocytotic markers such as transferrin, Rab5 and Caveolin, but localizes mainly in the cytoplasm and not in vesicles as observed by electron microscopy. The results are in perfect agreement with the fact that efficiency of CADY:siRNA mediated knockdown of the target gene is not altered in the presence of endocytotic inhibitors [24].

CADY:siRNA entry is controlled by the ability of the peptide to interact directly with the lipid moiety of the cell membrane and to induce a temporary membrane destabilization. Several studies have reported that high concentration of CPPs may trigger direct translocation or physical endocytosis [14], [20], [21], [44]. In the case of amphipathic peptides forming stable nanoparticles with their cargoes, such as MPG [23], [45], PEP [46] and now CADY (this work), clustering of the peptides around the cargo induces a high local CPP concentration at the cell membrane which favors cellular uptake through a mechanism independent of endocytosis even at low concentrations. The major driving force of CADY:siRNA cellular uptake is associated with structural dynamics and polymorphism of the peptide. Secondary structure as well as structural polymorphism of CPPs play a major role in cellular uptake of CPP/cargo complexes and in the balance between efficiency and toxicity as also reported for antimicrobial peptides [39]–[41], [47]. In contrast to other amphipathic peptides (MPG or PEP-1) that specifically interact with charged phospholipids and require an intact membrane potential to enter the cell [20], [37], CADY inserts spontaneously into monolayers containing either uncharged or charged phospholipids, irrespective of cholesterol concentration [28], which explains why membrane potential is not required to favour membrane crossing. Fura experiments demonstrated that CADY induces temporary membrane disorganization which is followed by rapid resealing leaving the cells intact and biologically active. This is in perfect agreement with the CPP's associated increase in membrane fluidity, and their ability to trigger membrane repair response [20], [39], [40], [48].

## Materials and Methods

### Peptide synthesis and siRNA

CADY peptide (GLWRALWRLLRSLWRLLWKA-cya) was synthesized as described previously [24]. CADY peptide was resuspended at a concentration of 2 mg/ml in water containing 2% dimethylsulfoxide, sonicated for 10 minutes and further diluted in water to a 100 µM stock solution. The siRNA and fluorescently labeled siRNA (5′-FAM) were obtained from Eurogentec (Belgium). The different sequences are as follows: for anti-GAPDH: 5′ -CAUCAUCCCUGCCUCUACUTT- 3′ (sense strand) and for anti-CICB1: 5′ –GAAAUGUACCCUCCAGAAATT 3′ (sense strand). The stock concentration of siRNA was prepared at 5 µM in 50 mM Tris, 0,5 mM EDTA buffer.

### Cell culture

The human cervical carcinoma cell line HeLa was obtained from ATCC. The Chinese hamster ovary cell lines CHO^WT^, CHO^HS−/−^ and CHO^gl−/−^ were a kind gift from Pr. U. Langel. All media were obtained from Gibco. HeLa cells were maintained as monolayer cultures in Dulbecco's modified Eagle's medium (DMEM) Glutamax supplemented with 10% fetal calf serum (FCS) and 1% antibiotics. CHO cells were maintained as monolayer cultures in F-12 supplemented with 10% FCS and 2 mM L-glutamin.

### CADY-mediated siRNA knock down analysis by Western blotting

CADY:siRNA complexes were formed at 37°C for 30 minutes in 0,5×PBS at a concentration of 9600 nM and 240 nM, respectively. In a 35 mm dish, HeLa cells at 60% confluency were overlaid with 200 µl preformed complexes, incubated for 5 minutes, prior addition of 400 µl DMEM. After 4 hours of incubation, 1 ml of media containing 16% FCS was added. Cells were harvested by trypsination 48 hours after transfection and lyzed in buffer containing 50 mM Tris-HCl pH 7.5, 150 mM NaCl, 2 mM EDTA, 0.1% NP40 and 0.1% deoxycholate including 1×Complete Protease Inhibitor Cocktail (Roche). Cell lysates were kept on ice for 30 minutes, mixed every 5 minutes and centrifuged at +4°C for 15 minutes at 10,000*g*. Supernatants were collected and protein concentrations were determined using the Bradford assay. 10 µg cell extracts were separated by 12% sodium dodecyl sulfate-polyacrylamide gel. Proteins were transferred onto a nitrocellulose membrane, blocked in PBS+4% milk for 1 hour, followed by overnight incubation at +4°C with rabbit anti-actin (Sigma) and mouse anti-GAPDH (Santa Cruz). After washes in PBS+ 0.05%Tween, the membrane was incubated either with anti-rabbit-HRP and anti-mouse-HRP (GE Healthcare) followed by chemiluminescence detection (Perkin Elmer) or fluorescent detection with anti-rabbit-DyLight800 and anti-mouse-DyLight680 (Thermo Scientific) followed by detection using Odyssey (Li-Cor Biosciences).

### FACS analysis

In a 35 mm dish, cells were overlaid with 200 µl pre-formed CADY:siGAPDH-FITC complexes (4800 nM and 240 nM respectively in 0,5×PBS), incubated for 5 minutes, and then 400 µl of DMEM were added. After 1,5 hours of incubation, cells were washed with PBS, trypsinized for 5 minutes, washed and resuspended in PBS containing propidium iodide (2,5 µg/ml). FITC and propidium iodide fluorescence were immediately measured by flow cytometry using Facscalibur (Becton Dickinson) by acquiring 50000 cells.

### Co-staining of endosomal and lysosomal markers with fluorescently labeled siRNA

CADY:siRNA (5′- FAM) complexes were formed at 37°C for 30 minutes in 0,5×PBS at a concentration of 960 nM and 240 nM, respectively. In a 15 mm dish with a glass cover slip, HeLa cells were treated with lysotracker, CtB or transferrin at the time of transfection, either followed by analysis of live cells, or by fixation in 4% paraformaldehyde at indicated timepoints. Labeling of Rab5 and caveolin positive endosomes using mouse anti-rab5 and rabbit anti-caveolin (BD Bioscience) were performed post-fixation after membrane perforation using 0,5% TritonX-114. Cells were analysed using a Zeiss Axioplan2/LSM 510 META Confocal microscope.

### Measurement of intracellular calcium levels

30000 HeLa cells were seeded on a 35 mm glass bottom fluorodish the day before measurement. Cells were incubated with 5 µM FURA-2AM (Invitrogen)+0,08% pluronic (Invitrogen) in HEPES-Krebs-Ringer (HKR) buffer (125 mM NaCl, 5 mM KCl, 1.2 mM MgSO_4_, 1 mM CaCl_2_,×2H_2_O, 1.2 mM KH_2_PO_4_, 25 mM Hepes, 6 mM Glucose pH 7.4) for 1 hour. The medium was replaced with HKR buffer or HKR buffer without calcium and incubated further for 1 hour. 20 nM siRNA complexed to CADY at a 1∶20 molar ratio was added then the ratio of fluorescence emission at 510 nm following excitation at 340 nm and 380 nm was measured using an inverted Zeiss Axiovert 200 microscope equipped with an LCI plan-neofluar 25×/0,8 objective and a Cool SNAP CCD camera.

### Visualization of siRNA nanoparticles by transmission electron microscopy

The thiol group at 5′ end of siRNA was tagged with nanogold (NG) cluster (Monomaleimido Nanogold, Nanoprobes, NY, d 1.4 nm) and the conjugate purified as described earlier [49]. NG-labeled siRNA was complexed with CADY as described for FAM-siRNA above and HeLa cells incubated with the resulting nanoparticles for 30 min or 2 h. The specimens were fixed with glutaraldehyde and the nanogold label on siRNA was revealed as described earlier for peptide [49].

## Supporting Information

Figure S1 **Cytotoxicity in CADY transfected cells.** HeLa cells were pre-incubated for 30 mins at 37°C (**A**) or 4°C (**B**) prior to addition of 80 nM FITC-labeled siRNA complexed to CADY at a 1∶20 molar ratio, followed by 1.5 hr incubation at indicated temperatures. Cells were washed, trypsinized, stained with propidium iodide (PI) and analysed by FACS. 11% and 14% of non transfected and transfected cells respectively were PI positive at 37°C. 24% and 28% of non transfected and transfected cells respectively were PI positive at 4°C.
(TIF)
