# Supplementary figures and images for: Direct Translocation as Major Cellular Uptake for CADY Self-Assembling Peptide-Based Nanoparticles
Source: PLoS One. 2011 Oct 5;6(10):e25924. doi: 10.1371/journal.pone.0025924 (PMC3187819; doi:10.1371/journal.pone.0025924)

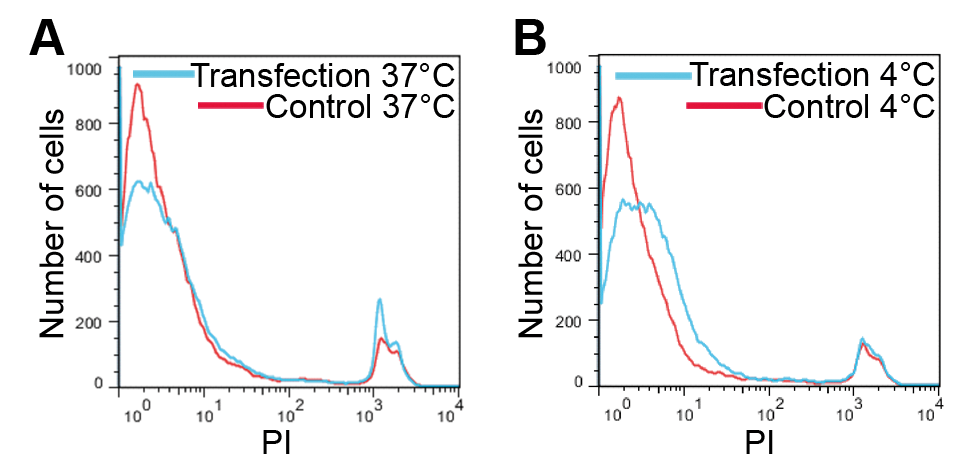

Supplement: Figure S1 — Cytotoxicity in CADY transfected cells. HeLa cells were pre-incubated for 30 mins at 37°C (A) or 4°C (B) prior to addition of 80 nM FITC-labeled siRNA complexed to CADY at a 1∶20 molar ratio, followed by 1.5 hr incubation at indicated temperatures. Cells were washed, trypsinized, stained with propidium iodide (PI) and analysed by FACS. 11% and 14% of non transfected and transfected cells respectively were PI positive at 37°C. 24% and 28% of non transfected and transfected cells respectively were PI positive at 4°C. (TIF) [file pone.0025924.s001.tif]
